# Supplementary material for: ACE2 N-glycosylation modulates interactions with SARS-CoV-2 spike protein in a site-specific manner
Source: Commun Biol. 2022 Nov 5;5:1188. doi: 10.1038/s42003-022-04170-6 (PMC9637154; doi:10.1038/s42003-022-04170-6)
Supplement: Supplementary file 2 — Supplementary Information FINAL [file 42003_2022_4170_MOESM2_ESM.pdf]

Supplementary Information

ACE2 *N*-glycosylation modulates interactions with SARS-CoV-2 spike protein in a site-specific manner

Ayana Isobe[1,8], Yasuha Arai[1,8], Daisuke Kuroda[2,8] , Nobuaki Okumura[3], Takao Ono[4], Shota Ushiba[5], Shin-ichi Nakakita[6], Tomo Daidoji[1], Yasuo Suzuki[7], Takaaki Nakaya[1], Kazuhiko Matsumoto[4], Yohei Watanabe[1*]

[1]Department of Infectious Diseases, Kyoto Prefectural University of Medicine, Kyoto 602-8566, Japan

[2]Research Center for Drug and Vaccine Development, National Institute of Infectious Diseases, Tokyo 162-8640, Japan.

[3]Institute for Protein Research, Osaka University, Osaka 565-0871, Japan

[4]SANKEN, Osaka University, Osaka 567-0047, Japan

[5]Murata Manufacturing Co., Ltd., Kyoto 617-8555, Japan

[6]Division of Functional Glycomics, Kagawa University, Kagawa 761-0793, Japan

[7]Department of Medical Biochemistry, School of Pharmaceutical Sciences, University of Shizuoka, Shizuoka 422-8526, Japan

[8]These authors contributed equally

[*]Correspondence: nabe@koto.kpu-m.ac.jp (Y. Watanabe)

Supplementary Figures:

Supplementary Fig. 1 Structural and conformational stability of the ACE2-RBD complex.

Supplementary Fig. 2 Uncropped and unedited blot image

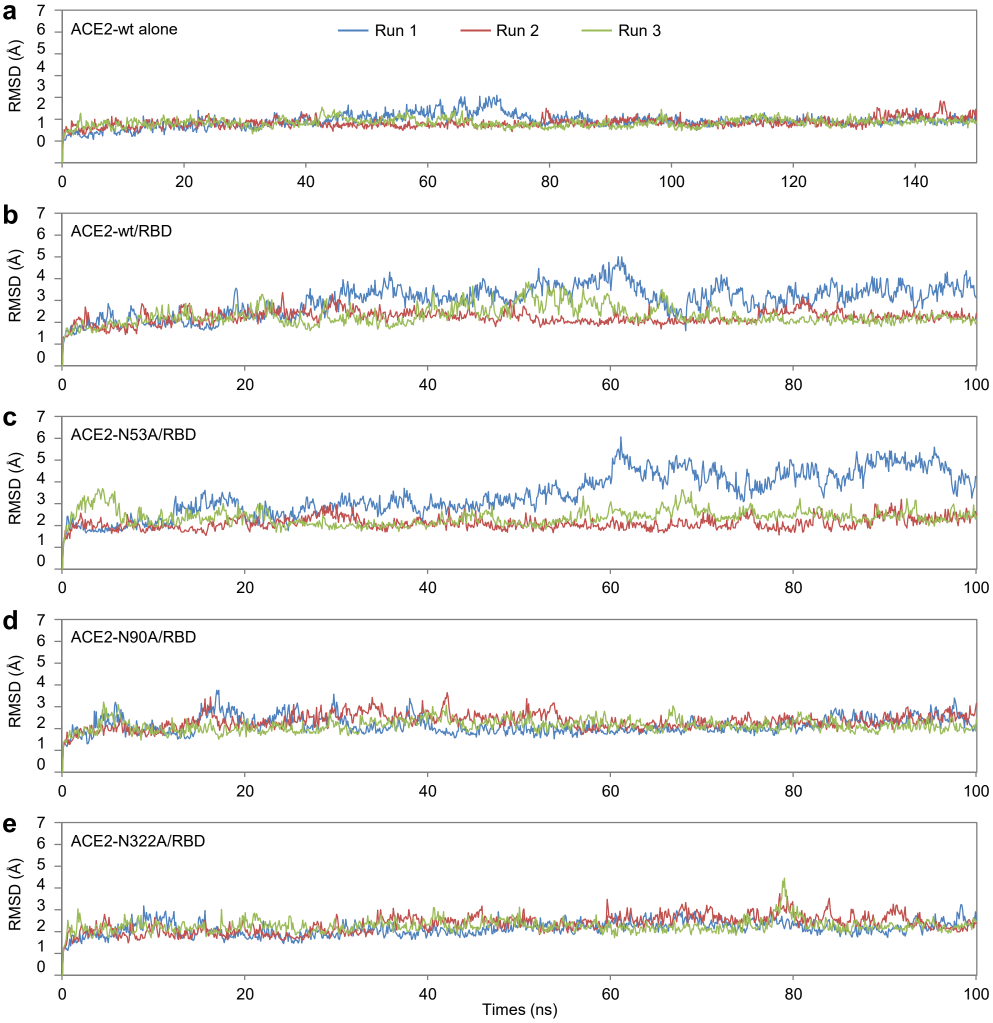

Supplementary Fig. 1. Structural and conformational stability of the ACE2-RBD complex.
Time trace of the Ca RMSD of the ACE2-RBD complex in three simulation runs. (a) ACE2-alone. (b) ACE2-wt/RBD complex. (c) ACE2-N53A/RBD complex. (d) ACE2-N90A/RBD complex. (e) ACE2-N322A/RBD complex. The complex was first superimposed onto the initial structure using the Cα atoms and then the RMSD was calculated.

related to Fig. 4a

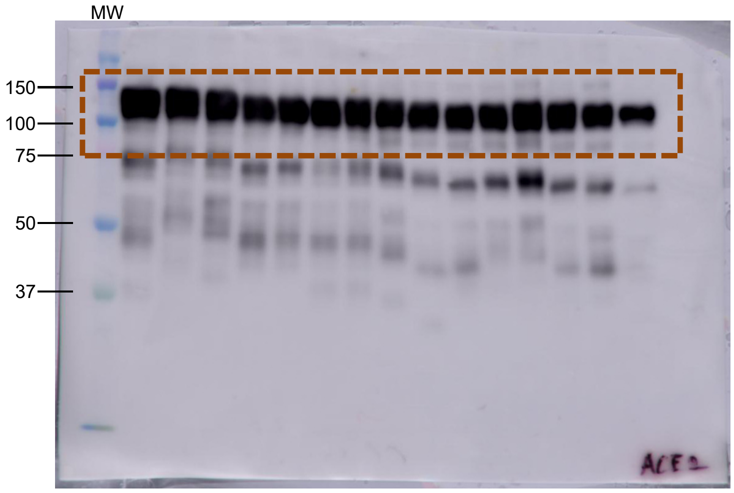

Supplementary Fig. 2. Uncropped and unedited blot image.
