# Supplementary material for: ACE2 N-glycosylation modulates interactions with SARS-CoV-2 spike protein in a site-specific manner
Source: Commun Biol. 2022 Nov 5;5:1188. doi: 10.1038/s42003-022-04170-6 (PMC9637154; doi:10.1038/s42003-022-04170-6)
Supplement: Supplementary file 3 — Description of Additional Supplementary Data [file 42003_2022_4170_MOESM3_ESM.docx]

**Description of Additional Supplementary Files**

**File name:** Supplementary Data 1

**Description:** The source data behind the graphs in the paper

**File name:** Supplementary Movie 1

**Description:** Molecular dynamics simulation of ACE2 bound to S-RBD. ACE2 and S-RBD are shown in light gray and green, respectively. N53, N90 and N322 residues on ACE2 are shown in cyan, yellow and orange, respectively and glycans at the three sites are shown in black.
