# Supplementary material for: ACE2 N-glycosylation modulates interactions with SARS-CoV-2 spike protein in a site-specific manner
Source: Commun Biol. 2022 Nov 5;5:1188. doi: 10.1038/s42003-022-04170-6 (PMC9637154; doi:10.1038/s42003-022-04170-6)
Supplement: Supplementary file 6 — Reporting Summary-New [file 42003_2022_4170_MOESM6_ESM.pdf]

nature portfolio

Corresponding author(s): Watanabe

Last updated by author(s): Oct 25, 2022

Reporting Summary

Nature Portfolio wishes to improve the reproducibility of the work that we publish. This form provides structure for consistency and transparency in reporting. For further information on Nature Portfolio policies, see our Editorial Policies and the Editorial Policy Checklist.

Statistics

For all statistical analyses, confirm that the following items are present in the figure legend, table legend, main text, or Methods section.

n/a	Confirmed	
☐	☒	The exact sample size (*n*) for each experimental group/condition, given as a discrete number and unit of measurement
☐	☒	A statement on whether measurements were taken from distinct samples or whether the same sample was measured repeatedly
☐	☒	The statistical test(s) used AND whether they are one- or two-sided<br>*Only common tests should be described solely by name; describe more complex techniques in the Methods section.*
☐	☒	A description of all covariates tested
☐	☒	A description of any assumptions or corrections, such as tests of normality and adjustment for multiple comparisons
☐	☒	A full description of the statistical parameters including central tendency (e.g. means) or other basic estimates (e.g. regression coefficient) AND variation (e.g. standard deviation) or associated estimates of uncertainty (e.g. confidence intervals)
☐	☒	For null hypothesis testing, the test statistic (e.g. *F*, *t*, *r*) with confidence intervals, effect sizes, degrees of freedom and *P* value noted<br>*Give P values as exact values whenever suitable.*
☒	☐	For Bayesian analysis, information on the choice of priors and Markov chain Monte Carlo settings
☒	☐	For hierarchical and complex designs, identification of the appropriate level for tests and full reporting of outcomes
☒	☐	Estimates of effect sizes (e.g. Cohen's *d*, Pearson's *r*), indicating how they were calculated

Our web collection on statistics for biologists contains articles on many of the points above.

Software and code

Policy information about availability of computer code

Data collection	ECLIPSE Ti2 inverted fluorescence microscope (Nikon)<br>BLItz System (SARTORIUS/ForteBio)<br>Amersham Imager 680 (GE Healthcare)<br>Auto maX MALDI-TOF MS (Bruker)
Data analysis	GraphPad Prism 6<br>Excel<br>ForteBio Data Analysis 9.0 software<br>Amersham Imager 680 Analysis software<br>flexAnalysis<br>Origin 9.1 software<br>GROMACS 2018.6

For manuscripts utilizing custom algorithms or software that are central to the research but not yet described in published literature, software must be made available to editors and reviewers. We strongly encourage code deposition in a community repository (e.g. GitHub). See the Nature Portfolio guidelines for submitting code & software for further information.

Data

Policy information about availability of data

All manuscripts must include a data availability statement. This statement should provide the following information, where applicable:

- Accession codes, unique identifiers, or web links for publicly available datasets
- A description of any restrictions on data availability
- For clinical datasets or third party data, please ensure that the statement adheres to our policy

The raw data in this study are available from the corresponding author with no restrictions upon reasonable request.

Human research participants

Policy information about studies involving human research participants and Sex and Gender in Research.

Reporting on sex and gender	n/a
Population characteristics	n/a
Recruitment	n/a
Ethics oversight	n/a

Note that full information on the approval of the study protocol must also be provided in the manuscript.

Field-specific reporting

Please select the one below that is the best fit for your research. If you are not sure, read the appropriate sections before making your selection.

☒ Life sciences ☐ Behavioural & social sciences ☐ Ecological, evolutionary & environmental sciences

For a reference copy of the document with all sections, see nature.com/documents/nr-reporting-summary-flat.pdf

Life sciences study design

All studies must disclose on these points even when the disclosure is negative.

Sample size	The sample sizes (n = 3) for virus infection and FRINT assay were chosen for applying statistical tests.
Data exclusions	We did not exclude specific sample(s) from analyses in our lab experiments.
Replication	Experiments were done at least two times as biological replicates.
Randomization	We did not use ramdomisation in our lab experimental designs.
Blinding	We did not use blinding in our lab experimental designs.

Reporting for specific materials, systems and methods

We require information from authors about some types of materials, experimental systems and methods used in many studies. Here, indicate whether each material, system or method listed is relevant to your study. If you are not sure if a list item applies to your research, read the appropriate section before selecting a response.

Materials & experimental systems

n/a	Involved in the study
☐	☒ Antibodies
☐	☒ Eukaryotic cell lines
☒	☐ Palaeontology and archaeology
☒	☐ Animals and other organisms
☒	☐ Clinical data
☒	☐ Dual use research of concern

Methods

n/a	Involved in the study
☒	☐ ChIP-seq
☒	☐ Flow cytometry
☒	☐ MRI-based neuroimaging

Antibodies

Antibodies used

For IF:
SARS-CoV-2 (COVID-19) Nucleocapsid antibody (Gene Tex, GTX135357, 1:1000)
SARS-CoV-1/2 Nucleocapsid antibody (Sigma-Aldrich, ZMS1075, 1:1000)
Goat anti-Rabbit IgG (H+L) Highly Cross-Adsorbed Secondary Antibody, Alexa Fluor 488 (Invitrogen, A-11034, 1:1000)
Goat anti-Mouse IgG (H+L) Highly Cross-Adsorbed Secondary Antibody, Alexa Fluor 488 (Invitrogten, A-11029, 1:1000)

For Western blot:
hACE2 antibody (Gene Tex, GTX101395, 5000)
Peroxidase Donkey Anti-Rabbit IgG (H+L) (Jackson Immuno Research 711-035-152, 1:5000)

Validation

Validation of all primary antibodies for the species and application was conducted by manufacturers prior to sale, and vlidation statments are available on the manufacturers' website.

Eukaryotic cell lines

Policy information about cell lines and Sex and Gender in Research

Cell line source(s)

Human 293T cells (RIKEN BRC, RCB2202)
Monkey Vero cells (RIKEN BRC, RCB0001)
Monkey Vero/TMPRSS2 cells (JCRB Cell Bank, JCRB1819)

Authentication

293T cells and Vero cells were authenticated by ATCC.
Vero/TMPRSS2 cells were authenticated by JCRB Cell Bank.

Mycoplasma contamination

All cell lines were regularly tested for mycoplasma contamination by using PCR and were confirmed to be mycoplasma-free.

Commonly misidentified lines
(See ICLAC register)

No commonly misidentified lines were used.
